# Intact and total insulin-like growth factor-binding protein-3 (IGFBP-3) levels in relation to breast cancer risk factors: a cross-sectional study

**DOI:** 10.1186/bcr2093

**Published:** 2008-05-09

**Authors:** Caroline Diorio, Jacques Brisson, Sylvie Bérubé, Michael Pollak

**Affiliations:** 1 McGill University, Breast Cancer Functional Genomics Group and McGill Centre for Bioinformatics, 3775 University Street, Montreal, QC H3A 2B4, Canada; 2 Université Laval, Département de médecine sociale et préventive, 2180 Chemin Sainte-Foy, Québec, QC G1K 7P4, Canada; 3 Unité de recherche en santé des populations, Centre hospitalier affilié universitaire de Québec, 1050 Chemin Sainte-Foy, Québec, QC G1S 4L8, Canada; 4 Centre des maladies du sein Deschênes-Fabia, Centre hospitalier affilié universitaire de Québec, 1050 Chemin Sainte-Foy, Québec, QC G1S 4L8, Canada; 5 Cancer Prevention Research Unit, Lady Davis Institute of the Jewish General Hospital and McGill University, Departments of Medicine and Oncology, 3755 Cote Ste-Catherine Road, Montreal, QC H3T 1E2, Canada

## Abstract

**Introduction:**

Levels of insulin-like growth factor (IGF)-I and its main binding protein (IGFBP-3) have been associated with breast cancer risk among premenopausal women. However, associations of IGFBP-3 levels with breast cancer risk have been inconsistent, possibly due to the different predominant forms of circulating IGFBP-3 (intact versus fragmented) that were measured in these studies. Here, we examine the association of breast cancer risk factors with intact and total IGFBP-3 levels.

**Methods:**

This cross-sectional study includes 737 premenopausal women recruited at screening mammography. Plasma intact and total IGFBP-3 and IGF-I levels were measured by enzyme-linked immunosorbent assay methods. Percent and absolute breast density were estimated using a computer-assisted method. The associations were evaluated using generalized linear models and Pearson (r) or Spearman (r_s_) partial correlation coefficients.

**Results:**

Means ± standard deviations of intact and total IGFBP-3 levels (ng/mL) were 1,044 ± 234 and 4,806 ± 910, respectively. Intact and total IGFBP-3 levels were correlated with age and smoking. Levels of intact IGFBP-3 were negatively correlated with waist-to-hip ratio (WHR) (r = -0.128; *P *= 0.0005), parity (r_s _= -0.078; *P *= 0.04), and alcohol intake (r = -0.137; *P *= 0.0002) and positively correlated with energy intake (r = 0.075; *P *= 0.04). In contrast, total IGFBP-3 levels were positively correlated with WHR (r = 0.115; *P *= 0.002), parity (r_s _= 0.089; *P *= 0.02), body mass index (BMI) (r = 0.115; *P *= 0.002), physical activity (r = 0.118; *P *= 0.002), and IGF-I levels (r = 0.588; *P *< 0.0001) and negatively correlated with percent or absolute breast density (r = -0.095; *P *= 0.01 and r = -0.075; *P *= 0.04, respectively).

**Conclusion:**

Our data show that associations of some breast cancer risk factors with intact levels of IGFBP-3 are different from those with total (intact and fragmented) IGFBP-3 levels. These findings suggest that different molecular forms of IGFBP-3 may bear different relations to premenopausal breast cancer risk.

## Introduction

Members of the insulin-like growth factor (IGF) family have been suggested to play a role in the occurrence of cancer at various sites, including the breast [1]. In particular, laboratory studies showed that IGF-I is able to exert mitogenic and antiapoptotic effects on normal and abnormal breast cells [2]. These results are consistent with the systematic reviews reporting that higher levels of IGF-I are associated with an increased breast cancer risk among premenopausal women (reviewed in [3]).

In contrast, associations of levels of total (intact and fragmented) IGF-binding protein-3 (IGFBP-3), the main binding protein of circulating IGF-I, with risk of breast cancer are conflicting and range from a protective association in some studies to an elevated risk in others (reviewed in [3]). Similarly, several studies have examined the association of breast cancer risk factors, such as personal characteristics, including lifestyle factors, and mammographic breast density, with total IGFBP-3 levels and found inconsistent results [4-13].

At the cellular level, IGFBP-3 has been found to either suppress or enhance the action of IGF-I and these effects are regulated, at least in part, by the presence of IGFBP proteases [14]. As a result, it has been proposed that the divergence in risk estimates among studies could be due to the predominant circulating forms of IGFBP-3 (intact versus fragmented) that have been measured. This notion was examined by Rinaldi and colleagues [15] in a population of young women, and their results suggested that high levels of functional IGFBP-3, which are composed of intact IGFBP-3 and some fragments of IGFBP-3, could be associated with a reduction of breast cancer risk (odds ratio [OR] = 0.54) whereas high levels of total IGFBP-3 could be associated with an increased risk of breast cancer (OR = 1.47). Thus, the variation in intact/functional versus total IGFBP-3 levels among subjects may differently modulate the risk of breast cancer.

The aim of this study was to examine whether circulating levels of intact IGFBP-3 and total IGFBP-3 were differently associated with several breast cancer risk factors, including mammographic breast density, a strong and independent breast cancer risk indicator [16]. To our knowledge, no other study has examined these associations with different molecular forms of IGFBP-3.

## Materials and methods

### Study population and recruitment procedures

Details of the study design and methods have been published elsewhere [6]. Briefly, the study subjects for the present analysis were premenopausal women who received a screening mammogram between February and December 2001 at the Clinique radiologique Audet (Québec, QC, Canada). The study focused only on premenopausal women because in previous analyses of our data the associations of total IGF-I and IGFBP-3 levels with breast density were observed only among these women [6]. Women were considered premenopausal if they had at least one natural menstrual cycle within 12 months or were younger than 48 years old (if a nonsmoker) or 46 years old (if a smoker) after hysterectomy without bilateral oophorectomy [17]. Exclusion criteria included the following: diabetes mellitus; dwarfism/acromegaly; thyroid, adrenal or hepatic disease; pregnancy; use of hormonal derivatives in the last 3 months before mammography; ever use of tamoxifen or raloxifene; personal history of cancer; or breast surgery.

A total of 787 premenopausal women were found to be eligible. Among these women, 2 declined participation, 2 could not provide film mammograms, and 10 had incomplete answers for some breast cancer risk factors. In the remaining 773 women, 36 did not give authorization for blood banking of samples for further study. Therefore, a total of 737 women were included in the present analysis. This study was reviewed and approved by the research ethics committee of the Centre hospitalier affilié universitaire de Québec.

### Data collection

At the radiology clinic, a trained nurse measured the women's weight (kilograms), height (centimetres), and waist and hip circumferences (centimetres) and collected 20 mL of blood. Known or suspected breast cancer risk factors were documented by a telephone interview and included reproductive and menstrual history, family history of breast cancer, personal history of breast biopsies, past use of contraceptives and hormone replacement therapy, smoking status, alcohol intake, education, and physical activity. The levels of physical activity in metabolic equivalent-hours per week were assessed using the validated and reproducible Nurses' Health Study II Activity and Inactivity Questionnaire [18]. Finally, each woman completed a validated [19] and self-administered semiquantitative food frequency questionnaire (97GP copyrighted at Harvard University, Boston, MA, USA). Food intake data obtained through the questionnaire were translated into nutrient intake, including energy intake (kilocalories per day), at the Channing Laboratory of Harvard University.

All mammograms were scanned at 260 μm/pixel with a Kodak Lumiscan85 digitizer (Eastman Kodak Company, Rochester, NY, USA). Then, for each woman, the proportion of the breast showing tissue density (percent density in percentage) and the absolute amount of dense tissue (absolute density in square centimetres) were assessed by one trained author (CD) from the craniocaudal view of a randomly chosen breast. This assessment was performed using a computer-assisted method without any information on the women [20]. Variability in the assessment of breast density was as follows: the within-batch intraclass correlation coefficients (n = 210 duplicate images) were 0.98 and 0.98 and the between-batch coefficients of variation (n = 10 images repeated 21 times) were 4% and 5% for percent and absolute breast density measurements, respectively.

At the time of blood collection, blood constituents were rapidly aliquoted and stored at -80°C until analysis. Under the supervision of one of us (MP), plasma levels of total IGF-I, total IGFBP-3, and intact IGFBP-3 (ng/mL) were blindly assayed by enzyme-linked immunosorbent assay with reagents from Diagnostic Systems Laboratories (Webster, TX, USA). Detailed methods have been published elsewhere [21]. In the present study, a proxy of fragmented IGFBP-3 levels was obtained by subtracting intact IGFBP-3 levels from the total levels of IGFBP-3. These calculated levels of fragmented IGFBP-3 are based on the assumptions that (a) the assay used to measure total IGFBP-3 captures all IGFBP-3 fragments, including the intact form, and (b) the assay used to measure intact IGFBP-3 captures all intact IGFBP-3. The intra-batch coefficients of variation (4 samples per batch of 39 samples for a total of 46 batches) were 10.5% and 13.2% and the between-batch coefficients of variation were 7.9% and 10.5% for total IGF-I and total IGFBP-3, respectively [6]. The intra- and between-batch coefficients of variation (4 samples per batch of 39 samples for a total of 22 batches) were 8.2% and 9.4%, respectively, for intact IGFBP-3.

### Statistical methods

Associations of breast cancer risk factors with continuous levels of intact, fragmented, and total IGFBP-3 were evaluated with the Spearman or Pearson correlation coefficients whether factors were treated as dichotomous or continuous variables, respectively. Multivariate-adjusted mean IGFBP-3 levels were assessed according to categories of variables (usually quartiles) using generalized linear models. The same approach was used to obtain mean mammographic breast density by quartiles of IGFBP-3 levels. However, absolute breast density was square-root-transformed to normalize its skewed distribution, and means are presented as back-transformed values. All statistical analyses were carried out using the SAS software system (SAS Institute Inc., Cary, NC, USA). Statistical significance was based on two-sided *P *values.

## Results

Characteristics of premenopausal women are described in Table 1. The mean value ± standard deviation for intact IGFBP-3 levels (1,044 ± 234 mg/mL) was clearly lower than that of total IGFBP-3 levels (4,806 ± 910 mg/mL). The Pearson correlations of intact IGFBP-3 levels with fragmented and total IGFBP-3 levels were in the opposite direction (r = -0.119; *P *= 0.001 and r = 0.139; *P *= 0.0002, respectively). The correlation between levels of fragmented and total IGFBP-3 was very high (r = 0.967; *P *< 0.0001).

**Table 1:** Description of the study population (n = 737)

	Mean ± SD or Percentage
Growth factors	
Total IGF-I, ng/mL	224.0 ± 63.7
Intact IGFBP-3, ng/mL	1,044 ± 234
Fragmented IGFBP-3, ng/mL	3,762 ± 908
Total IGFBP-3, ng/mL	4,806 ± 910
Characteristics	
Age at mammography, years	46.8 ± 4.6
Body mass index, kg/m^2^	25.2 ± 4.5
Waist-to-hip ratio	0.78 ± 0.06
Alcohol intake, drinks/week	3.4 ± 3.8
Energy intake, kilocalories/day	1,905 ± 514
Physical activity, MET-hours/week	27.2 ± 22.2
Parity, parous percentage	75.9
Smoking, current percentage	14.9
Mammographic breast density	
Percent density, percentage	42.4 ± 24.4
Absolute density, cm^2^	47.0 ± 28.8

IGF, insulin-like growth factor; IGFBP-3, insulin-like growth factor-binding protein-3; MET, metabolic equivalent; SD, standard deviation.

The multivariate-adjusted correlation of women's characteristics with IGFBP-3 levels varied according to the molecular form of the protein (Table 2). Levels of intact IGFBP-3 were negatively correlated with waist-to-hip ratio (WHR) (r = -0.128; *P *= 0.0005), alcohol intake (r = -0.137; *P *= 0.0002), and parity (r_s _= -0.078; *P *= 0.04) and positively correlated with energy intake (r = 0.075; *P *= 0.04); no correlation was observed with body mass index (BMI), physical activity, and IGF-I levels. In contrast, fragmented and total IGFBP-3 levels were both positively correlated with BMI (r = 0.124; *P *= 0.0008 and r = 0.115; *P *= 0.002), WHR (r = 0.156; *P *< 0.0001 and r = 0.115; *P *= 0.002), physical activity (r = 0.097; *P *= 0.009 and r = 0.118; *P *= 0.002), parity (r_s _= 0.110; *P *= 0.003 and r_s _= 0.089; *P *= 0.02), and IGF-I levels (r = 0.588; *P *< 0.0001 and r = 0.588; *P *< 0.0001). Moreover, fragmented IGFBP-3 levels were positively correlated with alcohol intake and negatively correlated with energy intake. Finally, intact, fragmented, or total IGFBP-3 levels were all positively correlated with age and negatively correlated with smoking (although the correlation was not statistically significant for smoking with intact IGFBP-3 levels, *P *= 0.09).

**Table 2:** Means and correlations of IGFBP-3 levels with IGF-I levels and women's characteristics

		Adjusted means of IGFBP-3 levels^a^		
		
	Number	Intact, ng/mL	Fragmented, ng/mL	Total, ng/mL
Total IGF-I, ng/mL				
≤179.6	185	1,051	3,081	4,132
179.7–218.1	184	1,040	3,587	4,627
218.2–260.7	184	1,033	3,935	4,968
> 260.7	184	1,051	4,451	5,502
r^b ^(*P*)		0.010 (0.78)	0.554 (< 0.0001)	0.555 (< 0.0001)
r^b ^(*P*)^a^		0.023 (0.53)	0.588 (< 0.0001)	0.588 (< 0.0001)
Age at mammography, years				
≤44	221	1,007	3,673	4,680
45–47	168	1,025	3,746	4,770
48–50	200	1,062	3,821	4,883
> 50	148	1,096	3,835	4,931
r^b ^(*P*)		0.083 (0.02)	0.028 (0.46)	0.049 (0.19)
r^b ^(*P*)^a^		0.113 (0.002)	0.104 (0.005)	0.139 (0.0002)
Body mass index, kg/m^2^				
≤22.0	186	1,071	3,680	4,750
22.1–24.4	187	1,057	3,671	4,728
24.5–27.4	183	1,003	3,771	4,775
> 27.4	181	1,044	3,932	4,976
r^b ^(*P*)		-0.070 (0.06)	0.153 (< 0.0001)	0.134 (0.0003)
r^b ^(*P*)^a^		-0.024 (0.52)	0.124 (0.0008)	0.115 (0.002)
Waist-to-hip ratio				
≤0.740	184	1,106	3,620	4,726
0.741–0.780	196	1,062	3,708	4,770
0.781–0.820	185	987	3,685	4,671
> 0.820	172	1,017	4,061	5,078
r^b ^(*P*)		-0.139 (0.0001)	0.188 (< 0.0001)	0.152 (< 0.0001)
r^b ^(*P*)^a^		-0.128 (0.0005)	0.156 (< 0.0001)	0.115 (0.002)
Alcohol intake, drinks/week				
Non-drinkers	53	1,080	3,691	4,771
≤1.0	234	1,066	3,716	4,781
1.1–4.0	238	1,063	3,734	4,797
> 4.0	212	989	3,864	4,853
r^b ^(*P*)		-0.113 (0.002)	0.002 (0.96)	-0.027 (0.46)
r^b ^(*P*)^a^		-0.137 (0.0002)	0.079 (0.03)	0.035 (0.34)
Energy intake, kilocalories/day				
≤1,564.21	185	1,023	3,807	4,830
1,564.22–1,855.02	184	1,040	3,837	4,878
1,855.03–2,199.36	184	1,031	3,756	4,787
> 2,199.36	184	1,081	3,649	4,730
r^b ^(*P*)		0.058 (0.12)	-0.004 (0.92)	0.011 (0.76)
r^b ^(*P*)^a^		0.075 (0.04)	-0.080 (0.03)	-0.055 (0.14)
Physical activity, MET-hours/week				
≤10.79	185	1,036	3,647	4,683
10.80–22.27	184	1,052	3,737	4,789
22.28–36.64	184	1,033	3,783	4,815
> 36.64	184	1,055	3,884	4,939
r^b ^(*P*)		0. 077 (0.04)	0.031 (0.40)	0.050 (0.17)
r^b ^(*P*)^a^		0.069 (0.06)	0.097 (0.009)	0.118 (0.002)
Parity				
Nulliparous	178	1,079	3,616	4,695
Parous	559	1,033	3,809	4,842
r_s _^c ^(*P*)		-0.063 (0.09)	0.135 (0.0002)	0.121 (0.001)
r_s _^c ^(*P*)^a^		-0.078 (0.04)	0.110 (0.003)	0.089 (0.02)
Smoking status				
None/ex-smoker	627	1,048	3,791	4,839
Current	110	1,020	3,598	4,618
r_s _^c ^(*P*)		-0.094 (0.01)	-0.117 (0.001)	-0.129 (0.0004)
r_s _^c ^(*P*)^a^		-0.063 (0.09)	-0.090 (0.01)	-0.091 (0.01)

^a^Means and correlations are adjusted for variables in the table. ^b^Pearson correlation (r) between continuous variables; adjusted correlations are partial Pearson coefficients. ^c^Spearman correlation (r_s_) between dichotomous factor and continuous levels of IGFBP-3; adjusted correlations are partial Spearman coefficients. IGF, insulin-like growth factor; IGFBP-3, insulin-like growth factor-binding protein-3; MET, metabolic equivalent.

Weight, waist circumference, and hip circumference were negatively correlated with intact IGFBP-3 levels and positively correlated with fragmented and total IGFBP-3 levels after adjustment for age, alcohol and energy intakes, physical activity, parity, smoking, and IGF-I levels. These correlations were statistically significant, except for the one between intact IGFBP-3 levels and hip circumference (data not shown).

No association of age at menarche, number of full-term pregnancies, age at first full-term pregnancy, lactation, family history of breast cancer, number of breast biopsies, education, past use of oral contraceptives, past use of hormone replacement therapy, or height was observed with intact, fragmented, and total IGFBP-3 levels (data not shown). Moreover, all variables found to be correlated with intact, fragmented, or total IGFBP-3 levels remained statistically significant with comparable correlations after further adjustment for those factors (data not shown).

Table 3 shows correlations of mammographic breast density with intact, fragmented, and total IGFBP-3 levels. In unadjusted models, we found that intact IGFBP-3 levels were positively associated with percent breast density (mean percent breast density = 40.6, 41.6, 41.5, and 45.7; r = 0.075; *P *= 0.04) whereas fragmented (mean percent breast density = 47.1, 44.5, 40.4, and 37.4 %; r = -0.171; *P *< 0.0001) and total (mean percent breast density = 46.3, 45.6, 39.8, and 37.6 %; r = -0.151; *P *< 0.0001) IGFBP-3 levels were negatively associated with percent breast density. Negative associations of intact (mean absolute breast density = 48.2, 46.0, 47.0, and 46.7 cm^2^; r = -0.010; *P *= 0.79), fragmented (mean absolute breast density = 49.3, 48.3, 46.6, and 43.7 cm^2^; r = -0.092; *P *= 0.01), and total (mean absolute breast density = 50.2, 49.4, 45.0, and 43.3 cm^2^; r = -0.094; *P *= 0.01) IGFBP-3 levels with absolute breast density were observed before adjusting for confounders, although the association between intact IGFBP-3 levels and absolute breast density did not reach statistical significance. After adjustment for factors included in Table 1, fragmented and total IGFBP-3 levels were negatively correlated with percent (r = -0.105; *P *= 0.004 and r = -0.095; *P *= 0.01) and absolute (r = -0.066; *P *= 0.07 and r = -0.075; *P *= 0.04) breast density whereas levels of intact IGFBP-3 were not significantly correlated with either percent or absolute breast density. After further adjustment for age at menarche, number of full-term pregnancies, age at first full-term pregnancy, lactation, family history of breast cancer, number of breast biopsies, education, past use of oral contraceptives, past use of hormone replacement therapy, and height, these correlations were all slightly stronger, and the borderline negative correlation between absolute density and fragmented IGFBP-3 levels became statistically significant.

**Table 3:** Means and correlations of IGFBP-3 levels with mammographic breast density

	Adjusted means of percent density (percentage)^a^			Adjusted means of absolute density (cm^2^)^a,b^		
Levels of IGFBP-3	Intact^c^	Fragmented^d^	Total^e^	Intact^c^	Fragmented^d^	Total^e^
Quartile 1 (n = 185)	42.2	45.3	44.5	48.6	48.3	49.4
Quartile 2 (n = 184)	42.9	44.3	44.7	46.3	48.0	48.6
Quartile 3 (n = 184)	40.4	40.3	40.7	45.7	45.9	45.0
Quartile 4 (n = 184)	44.0	39.6	39.6	45.5	43.9	43.1
r^f ^(*P*)	0.075 (0.04)	-0.171 (< 0.0001)	-0.151 (< 0.0001)	-0.010 (0.79)	-0.092 (0.01)	-0.094 (0.01)
r^f ^(*P*)^a^	0.031 (0.40)	-0.105 (0.004)	-0.095 (0.01)	-0.029 (0.43)	-0.066 (0.07)	-0.075 (0.04)
r^f ^(*P*)^g^	0.022 (0.55)	-0.112 (0.003)	-0.104 (0.005)	-0.042 (0.26)	-0.078 (0.04)	-0.090 (0.02)

^a^Means and correlations are adjusted for age, body mass index, waist-to-hip ratio, alcohol intake, energy intake, physical activity, parity, smoking status, and total IGF-I levels. ^b^Means of absolute density are presented as back-transformed values; square-root-transformed absolute density is used in the correlation. ^c^Quartiles of intact IGFBP-3 are ≤883.80, 883.81 to 1,021.00, 1,021.01 to 1,170.50, and > 1,170.50 ng/mL. ^d^Quartiles of fragmented IGFBP-3 are ≤3,137.50, 3,137.51 to 3,662.50, 3,662.51 to 4,263.00, and > 4,263.00 ng/mL. ^e^Quartiles of total IGFBP-3 are ≤4,183.0, 4,183.1 to 4,695.0, 4,695.1 to 5,290.0, and > 5,290.0 ng/mL. ^f^Pearson correlation between continuous variables; adjusted correlations are partial Pearson coefficients. ^g^Correlations are adjusted for age, body mass index, waist-to-hip ratio, alcohol intake, energy intake, physical activity, parity, smoking status, total IGF-I levels, age at menarche, number of full-term pregnancies, age at first full-term pregnancy, lactation, family history of breast cancer, number of breast biopsies, education, past use of oral contraceptives, past use of hormone replacement therapy, and height.

## Discussion

Our data suggest that, among premenopausal women, the associations of some breast cancer risk factors with intact levels of IGFBP-3 are different from those with total (intact and fragmented) IGFBP-3 levels. Our data show that lower WHR, lower alcohol intake, and higher energy intake or nulliparity are associated with higher levels of intact IGFBP-3 whereas associations in the opposite direction are observed between these breast cancer risk factors and fragmented or total IGFBP-3 levels. Moreover, fragmented or total IGFBP-3 levels were negatively associated with mammographic breast density, one of the strongest known breast cancer risk factors, whereas no such association was seen with intact IGFBP-3. These findings suggest that different forms of IGFBP-3 may bear different relations to premenopausal breast cancer risk.

This is the first epidemiologic study to examine the association of intact and total IGFBP-3 measurements with a large set of breast cancer risk factors, including mammographic breast density. So far, two studies have reported on the association of different measurements of IGFBP-3 levels with the risk of breast cancer and found inconsistent results [15,22]. The first case-control study of 40 cases and 40 age- and race-matched controls among premenopausal and postmenopausal women failed to show any association of intact, fragmented, or total IGFBP-3 levels with the risk of breast cancer [22]. In contrast, recent data from the New York University Women's Health Study suggested that, among young women, high levels of functional IGFBP-3 could be associated with a reduction of breast cancer risk whereas high levels of total IGFBP-3 could be associated with an increased risk of breast cancer [15].

Mammographic breast density is a strong biomarker for breast cancer [16]. Contrary to our expectations, the associations of intact and total IGFBP-3 levels with breast density do not seem to mirror the IGFBP-3-breast cancer associations in the study of Rinaldi and colleagues [15]. However, both studies differ on several points regardless of the breast density/risk issue. In the study of Rinaldi and colleagues, women were younger and leaner and more were nulliparous and they had different mean levels of growth factors. Most importantly, the measurement of functional IGFBP-3 concentrations was assessed by ligand immunofunctional assay, which measured the forms of IGFBP-3 that are able to bind the IGF ligand. It has been shown that different lengths of IGFBP-3 fragments, including the first 97 residues (^1–97^IGFBP-3), are capable of binding IGFs, though with a lower affinity than intact IGFBP-3 [14,23]. In the present study, these ^1–97^IGFBP-3 fragments are not detected by the assay used [21] and, thus, such fragments are not contributing to our intact IGFBP-3 measurement. This difference between assays may explain, at least in part, the higher correlation between functional and total IGFBP-3 levels (r = 0.45) in the study of Rinaldi and colleagues.

Meanwhile, laboratory studies showed that different molecular forms of IGFBP-3 such as intact IGFBP-3, ^1–160^IGFBP-3, and ^1–95^IGFBP-3 fragments have different proliferative and apoptotic activities on cells [24,25]. It has been observed that, at the same concentration, ^1–160^IGFBP-3 fragments stimulate proliferation whereas ^1–95^IGFBP-3 fragments inhibit proliferation of prostate carcinoma cells [24]. Moreover, ^1–95^IGFBP-3 fragments were shown to induce morphological changes and apoptosis of breast carcinoma cells [25]. The ^1–95^IGFBP-3 fragments were suggested to inhibit, at least in part, the mitogenic signals resulting from IGF-I receptor activation [23,24,26,27]. These findings suggest that the proportions of ^1–95^IGFBP-3, ^1–160^IGFBP-3, and ^1–264^IGFBP-3 (intact IGFBP-3) relative to total IGFBP-3 are important, and methods should be developed to precisely measure each of these fragments since they may have different effects on target cells. Therefore, differences in the assay and the molecular forms of IGFBP-3 measurement may explain, to some extent, the inconsistency between the study of Rinaldi and colleagues [15] and the present study and the heterogeneity between studies evaluating total IGFBP-3 levels with the risk of breast cancer or its associated risk factors [3-13].

The major strengths of this study include the reliability of breast density measurements, the extensive information on breast cancer risk factors, and the relatively large sample size. However, this study also has some limitations. First, levels of fragmented IGFBP-3 were not assayed but derived from the difference between levels of total and intact IGFBP-3. This difference can be considered only as a proxy of all IGFBP-3 fragments. Therefore, associations observed with estimated fragmented IGFBP-3 levels have to be interpreted with caution. Nevertheless, since the association of several breast cancer risk factors with estimated fragmented IGFBP-3 was quite different from their association with intact IGFBP-3, our results suggest that further studies should measure levels of fragmented IGFBP-3 using specific assays and should examine intact and fragmented IGFBP-3 separately. It has been proposed that long-term storage may increase proteolytic activity and, therefore, increase levels of fragmented IGFBP-3 [28]. However, our results were essentially unchanged after further adjustment for the length of storage in the analysis. Second, laboratory measurements were performed on non-fasting blood samples. In a recent study, it has been suggested that fasting and fed state can affect levels of IGF-I and IGFBP-3 [29]. However, our results remain similar when models were further adjusted for the number of hours since the last meal. Third, blood collection was not timed with a specific phase of the menstrual cycle. However, the phase of cycle at the time of blood collection was associated with neither IGFBP-3 levels nor breast density. Moreover, further adjustment for menstrual cycle phase at the time of blood collection (and mammogram) had essentially no confounding effect in these data. Fourth, because an exploratory approach was used and multiple testing was carried out, we cannot totally exclude the possibility that some of the findings could be due to chance. Moreover, some of the observed correlations are weak with uncertain clinical significance. Therefore, these data need to be confirmed by other studies. Finally, the cross-sectional design of the study does not allow us to determine the temporality of the relation between IGFBP-3 levels and breast cancer risk factors.

## Conclusion

The associations of several breast cancer risk factors with IGFBP-3 levels vary in strength and even direction depending on the molecular form of IGFBP-3. These results suggest that different molecular forms of circulating IGFBP-3 (intact versus fragmented) may bear different relations to the risk of breast cancer and, possibly, of cancer at other sites. Further studies measuring intact, fragmented, and total IGFBP-3 would help to identify whether this molecule is a cancer risk factor, a preventive factor, or both.

## Abbreviations

BMI = body mass index; IGF = insulin-like growth factor; IGFBP-3 = insulin-like growth factor-binding protein-3; MET, metabolic equivalent; OR = odds ratio; WHR = waist-to-hip ratio.

## Competing interests

The authors declare that they have no competing interests.

## Authors' contributions

CD was involved in the study design, data collection, mammographic breast density assessments, statistical analysis, and drafting of the manuscript. JB and SB were involved in the study design, data collection and statistical analysis. MP was involved in the study design and performed the growth factor analyses. All authors contributed to revisions of the manuscript and read and approved the final manuscript.
